# Urban particulate matter down-regulates filaggrin via COX2 expression/PGE2 production leading to skin barrier dysfunction

**DOI:** 10.1038/srep27995

**Published:** 2016-06-17

**Authors:** Chiang-Wen Lee, Zih-Chan Lin, Stephen Chu-Sung Hu, Yao-Chang Chiang, Lee-Fen Hsu, Yu-Ching Lin, I-Ta Lee, Ming-Horng Tsai, Jia-You Fang

**Affiliations:** 1 Department of Nursing, Division of Basic Medical Sciences, Chang Gung University of Science and Technology, Chia-Yi, Taiwan; 2 Research Center for Industry of Human Ecology, Chang Gung University of Science and Technology, Kweishan, Taoyuan, Taiwan; 3 Pharmaceutics Laboratory, Graduate Institute of Natural Products, Chang Gung University, Kweishan, Taoyuan, Taiwan; 4 School of Traditional Chinese Medicine, Chang Gung University, Kweishan, Taoyuan, Taiwan; 5 Department of Dermatology, College of Medicine, Kaohsiung Medical University, Kaohsiung, Taiwan; 6 Department of Dermatology, Kaohsiung Medical University Hospital, Kaohsiung, Taiwan; 7 Center for Drug Abuse and Addiction, China Medical University Hospital, Taichung, Taiwan; 8 School of Medicine, College of Medicine, China Medical University, Taichung, Taiwan; 9 Department of Respiratory Care, Chang Gung University of Science and Technology, Chiayi Campus, Chiayi, Taiwan; 10 Department of Respiratory Care, Chang Gung University, Taoyuan, Taiwan; 11 Division of Pulmonary and Critical Care Medicine, Chang Gung Memorial Hospital, Chiayi, Taiwan; 12 Department of Pediatrics, Division of Neonatology and Pediatric Hematology/Oncology, Chang Gung Memorial Hospital, Yunlin, Taiwan; 13 Chinese Herbal Medicine Research Team, Healthy Aging Research Center, Chang Gung University, Kweishan, Taoyuan, Taiwan

## Abstract

We explored the regulation of filaggrin, cyclooxygenase 2 (COX2) and prostaglandin E2 (PGE2) expression induced by urban particulate matter (PM) in human keratinocytes. In addition, we investigated the signaling pathways involved in PM-induced effects on COX2/PGE2 and filaggrin. PMs induced increases in COX2 expression and PGE2 production, and decreased filaggrin expression. These effects were attenuated by pretreatment with COX2 inhibitor and PGE2 receptor antagonist, or after transfection with siRNAs of the aryl hydrocarbon receptor (AhR), gp91phox and p47phox. Furthermore, PM-induced generation of reactive oxygen species (ROS) and NADPH oxidase activity was attenuated by pretreatment with an AhR antagonist (AhRI) or antioxidants. Moreover, Nox-dependent ROS generation led to phosphorylation of ERK1/2, p38, and JNK, which then activated the downstream molecules NF-κB and AP-1, respectively. *In vivo* studies in PMs-treated mice showed that AhRI and apocynin (a Nox2 inhibitor) had anti-inflammatory effects by decreasing COX2 and increasing filaggrin expression. Our results reveal for the first time that PMs-induced ROS generation is mediated through the AhR/p47 phox/NADPH oxidase pathway, which in turn activates ERK1/2, p38/NF-κB and JNK/AP-1, and which ultimately induces COX2 expression and filaggrin downregulation. Up-regulated expression of COX2 and production of PGE2 may lead to impairment of skin barrier function.

In our modern era of rapid industrialization, exposure to air pollution, both at population and individual levels, is associated with adverse effects on human health^1^,^2^. Recent studies have demonstrated that exposure to airborne particulate matters (PMs) by inhalation correlates with pulmonary dysfunction, cardiovascular disease, and hepatic fibrogenesis^3^,^4^,^5^. PMs are complex mixtures of polyaromatic hydrocarbons, metals, organic toxins and biological materials that potentially trigger oxidative stress^6^,^7^,^8^. In addition to numerous epidemiologic studies that have demonstrated adverse effects of PMs on human health, they also induce allergic sensitization and provoke adaptive immune responses^9^,^10^,^11^. In addition, PMs have been found to stimulate production of pro-inflammatory cytokines, accelerate coagulation, increase the activity of endocrine systems, and contribute to neurotoxicity^12^,^13^,^14^,^15^. However, most of the studies on health-related effects of PMs have focused on respiratory and cardiovascular diseases.

PM-induced toxic effects in humans occur mainly through inflammatory and oxidative stress mechanisms. These processes are closely interlinked and both involve activation of a series of mediators of the nicotinamide adenine dinucleotide phosphate (NADPH)-oxidase (NOX) family, generation of reactive oxygen species (ROS), and up-regulation of some transcription factors within cell nuclei^16^,^17^,^18^. The skin is the largest organ of the human body and constitutes the outermost barrier that comes into direct contact with air pollutants. Filaggrin–which plays a key role in conferring keratinocytes with their physical strength via aggregation of keratin bundles–contributes to epidermal hydration and barrier function^19^,^20^. Filaggrin gene mutations can lead to downstream immunologic activation, and subsequent synthesis and secretion of specific IgE antibodies against absorbed allergens, predisposing to skin barrier abnormalities^21^. A variety of pro-inflammatory cytokines, including cyclooxygenase 2 (COX2) and prostaglandin E2 (PGE2), exert their biological effects through signaling cascades, leading to skin inflammation^22^,^23^. Several investigations have shown activation of COX2/PGE2/nuclear factor kappaB (NFκB) signaling and down-regulation of filaggrin in the skin of patients with atopic dermatitis^24^,^25^. However, the interactions between these two signaling pathways, and the way in which their effects coordinate to increase the risk of skin barrier dysfunction, remain largely unclear.

Although some epidemiological evidence has demonstrated adverse effects of PM exposure on the skin^11^,^26^, studies on the underlying mechanisms are sparse. Pan *et al*.^27^ recently found that PMs disrupt tight junctions in the stratum corneum, which may lead to skin barrier dysfunction and increased risk of drug over-absorption. Current evidence is not sufficient to establish whether PM exposure affects the expression of filaggrin. We hypothesized that PMs regulate the expression of filaggrin and COX2 in human keratinocytes, with accompanying antioxidant/inflammatory responses. In this study, we screened for changes in filaggrin and COX2-related elements, using *in vivo* and *in vitro* exposure models involving standard reference airborne PMs (standard reference material [SRM] 1649b, issued by the National Institute of Standards and Technology [NIST], USA; average diameter: 10.5 μm). We also examined: 1) if the effect of PMs on filaggrin expression are present in the skin, 2) if there is a relationship between exposure to PMs and release of various pro-inflammatory mediators and antioxidant responses, and 3) if histologic evidence of tissue damage is present following exposure and changes in gene reprogramming.

## Results

### PMs up-regulate COX2 expression and increase PGE2 production, but down-regulate filaggrin expression in HaCaT cells

**Figure 1:**
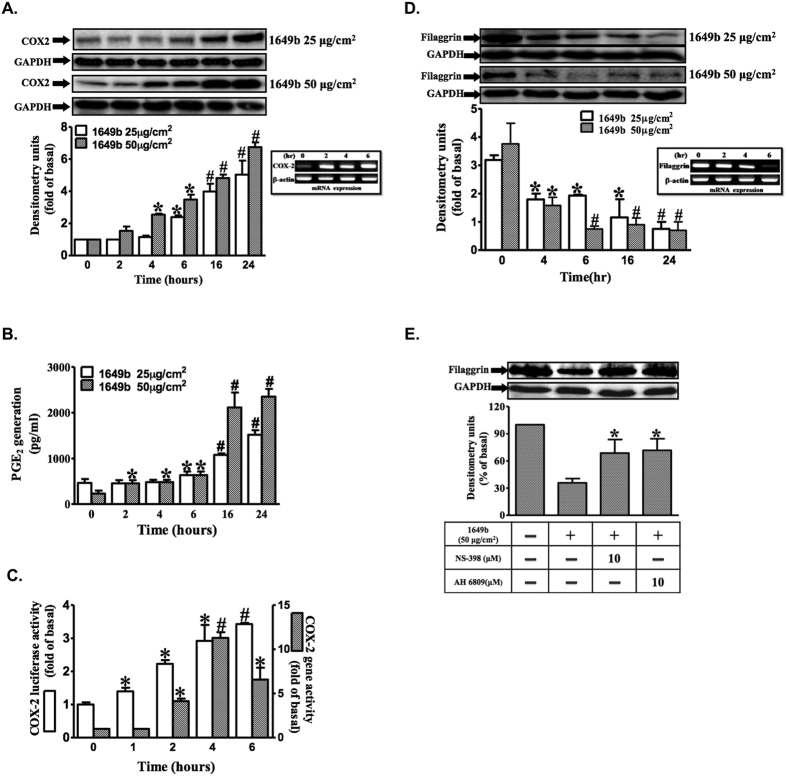
PMs up-regulate COX2 expression and increase PGE2 production, but down-regulate filaggrin expression in HaCaT cells. HaCaT cells were exposed to PMs (25 and 50 μg/cm^2^) for the indicated times (0, 2 h, 4 h, 6 h, 12 h and 24 h). (**A,D**) COX2 and filaggrin expression was determined by Western blotting and RT-PCR. Total glyceraldehyde 3-phosphate dehydrogenase (GAPDH) and β-actin were used as loading controls for protein and mRNA expressions, respectively. (**B**) PGE2 release was induced by PMs, and the conditioned media were collected from (**A**) to assay PGE2 level by ELISA as described in “Materials and methods” (n = 3 in each group; *P < 0.05, ^#^P < 0.01 compared with vehicle). (**C**) COX2 gene expression was analyzed by real-time PCR and by promoter luciferase activity as described in “Materials and methods”. (**E)** HaCaT cells were pre-incubated with NS-398 (10 μM) or AH6809 (10 μM) for 1 h and then exposed with PMs (50 μg/cm^2^) for 24 h. Filaggrin expression was determined by Western blotting. (**A–D**) Data are expressed as mean ± standard error of the mean, based on three independent experiments. ^*^P < 0.05, ^#^*P* < 0.01, compared with basal levels.

Western blot analysis revealed time- and dose-dependent increases in PM-induced COX2 expression. PMs (25 and 50 μg/cm^2^) induced significant increases in COX2 expression between 4 and 24 h post-treatment (Fig. 1A), and was associated with an increase in PGE2 production (Fig. 1B). PM exposure resulted in significant time-dependent increases in COX2 expression at the mRNA and promoter levels, with maximal response within 6 h (Fig. 1C). In addition, PMs induced down-regulation of filaggrin protein and mRNA in a time- and dose-dependent manner, based on respective Western blot and reverse-transcriptase polymerase chain reaction (RT-PCR) analyses (Fig. 1D). Moreover, pretreatment with NS-398, a selective COX2 inhibitor, or 6-isopropoxy-9-oxoxanthene-2-carboxylic acid (AH6809), a prostanoid EP1/EP2 receptor antagonist, significantly attenuated PM-induced filaggrin down-regulation (Fig. 1E). These findings indicate that PM-induced COX2/PGE2 up-regulation plays a direct role in the down-regulation of filaggrin. Moreover, results obtained using HaCaT cells suggest that PMs may cause skin barrier dysfunction by down-regulation of filaggrin, via a mechanism involving up-regulation of COX2/PGE2 expression.

### PMs induce filaggrin down-regulation and COX2/PGE2 expression via the aryl hydrocarbon receptor/Nox-dependent pathway

**Figure 2:**
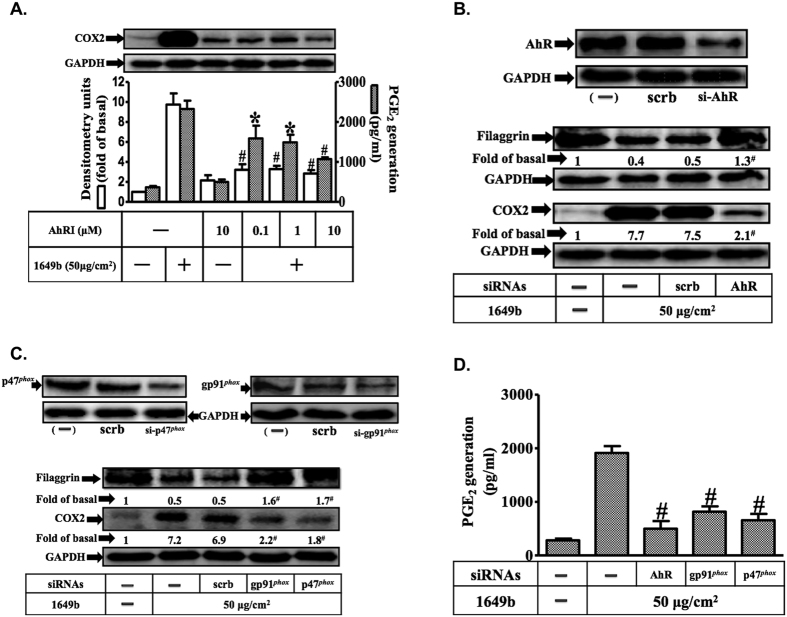
Aryl hydrocarbon receptor (AhR) and Nox2 are necessary for PMs-induced down-regulation of filaggrin and COX2/ PGE2 expression in HaCaT cells. Cells were pre-incubated with AhRI (an AhR antagonist) (0.1, 1, 10 μM) for 1 h and then exposed with PMs (50 μg/cm^2^) for 24 h. (**A**) Effects of AhRI (an AhR antagonist) on COX2 expression (open bars) and PGE2 release (shaded bars) were determined by Western blotting and ELISA as described in Fig. 1. (**B–D**) Effects of siRNAs of AhR, Nox2 (gp91^*phox*^) and p47^*phox*^ on filaggrin and COX2 protein expression and PGE2 release. (**A–D**) Data are expressed as mean ± standard error of the mean, based on three independent experiments. ^*^P < 0.05, ^#^*P* < 0.01, compared with basal levels.

We co-treated HaCaT cells with the selective aryl hydrocarbon receptor (AhR) antagonist, AhRI^28^, which significantly reduced PM-induced COX2 expression and PGE2 production (Fig. 2A). As shown in Fig. 2B–D, transfection with small interfering RNA (siRNA) of AhR resulted in significant knock down of AhR protein expression, and attenuated PM-induced COX2 expression, PGE2 production and filaggrin down-regulation. These effects were also observed after siRNA-induced suppression of Nox2 (gp91^*phox*^) and p47^*phox*^ expression. Taken together, these results suggest that PMs down-regulate filaggrin protein levels via AhR-mediated effects on Nox2 and COX2/PGE2 signaling in human keratinocytes.

### PMs-associated increases in NADPH oxidase activity and ROS generation contribute to increases in COX2 expression and PGE2 production

**Figure 3:**
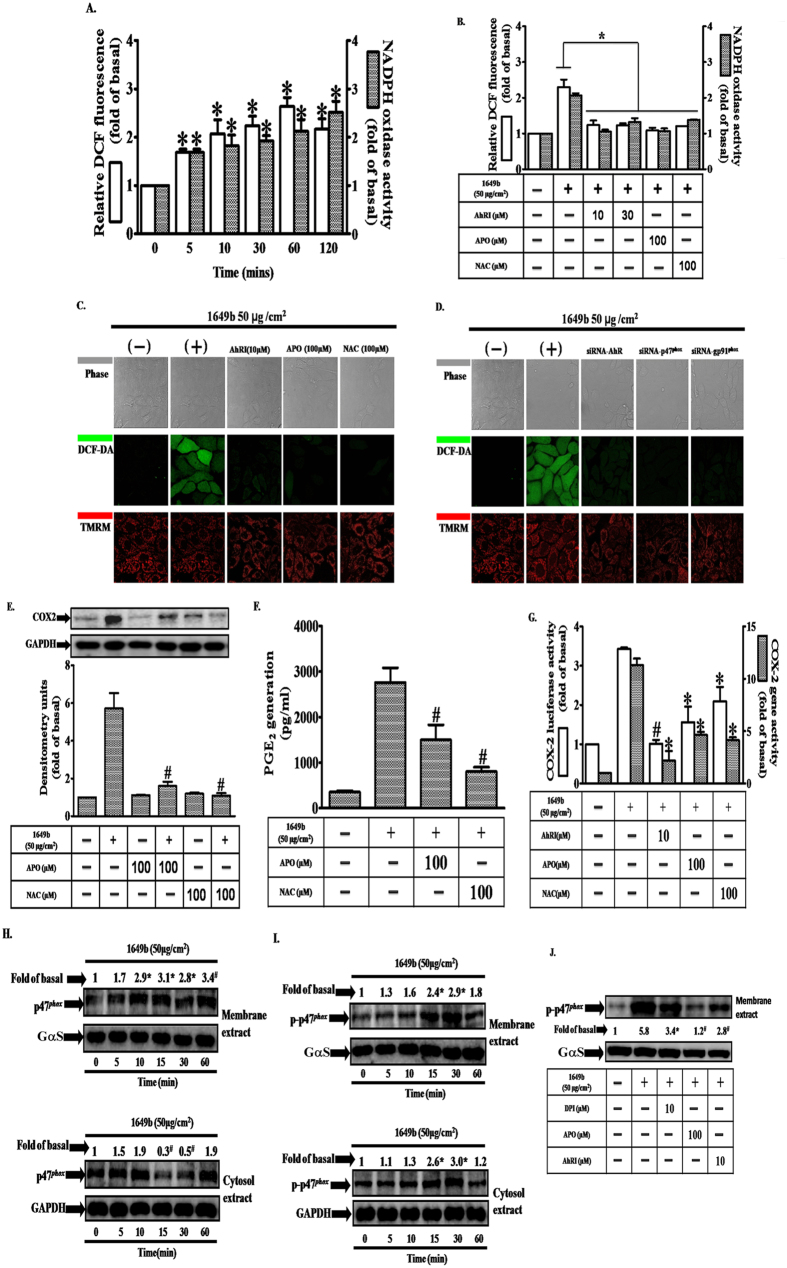
PMs induce NADPH oxidase activation, generation of reactive oxygen species (ROS) and p47^*phox*^ translocation in HaCaT cells. (**A**) Cells were exposed to PMs (50 μg/cm^2^) for the indicated times (0, 5′, 10′, 30′, 1 h and 2 h). (**B**) Cells were pre-incubated with AhRI (10 and 30 μM), APO (100 μM) or NAC (100 μM) for 1 h and then exposed to PMs (50 μg/cm^2^) for 24 h. Effects of PMs and antioxidants on ROS generation (open bars) and NADPH oxidase activity (shaded bars) were measured using DCF-DA fluorescence and lucigenin chemiluminescence as described in “Materials and methods” (n = 3 in each group; *P < 0.05 compared with vehicle), respectively. (**C**,**D**) Cells were transfected with siRNAs of the aryl hydrocarbon receptor (AhR), Nox2 (gp91^*phox*^) or p47^*phox*^ before exposure to PMs. ROS generation (green color) and mitochondrial membrane potential (red color) were evaluated by confocal microscopy. Effects of antioxidants (apocynin [APO] or N-acetylcysteine [NAC]) on cyclooxygenase 2 (COX2) protein expression (**E**), prostaglandin E2 (PGE2) production (**F**) and gene expression (**G**), determined by Western blot, ELISA, real-time PCR (shaded bars) and promoter activity (determined by luciferase assay) (open bars), respectively. (**H,I**) cells were exposed to PMs (50 μg/cm^2^) for the indicated times (0, 5′, 10′, 15′, 30′ and 1 h) and (**J**), cells were pre-incubated with AhRI (10 μM), APO (100 μM) or DPI (10 μM) for 1 h and then exposed to PMs (50 μg/cm^2^) for 30 min. Effects of antioxidants on p47^*phox*^ phospho rylation and translocation. Membrane (ME) and cytosolic (CE) fractions were prepared and subjected to Western blot using an anti-p47^*phox*^ or anti-phospho-p47^*phox*^ antibody. (**A–J**) Data are expressed as mean ± standard error of the mean, based on three independent experiments. ^*^P < 0.05, ^#^*P* < 0.01, compared with basal levels.

We examined the involvement of ROS and Nox in PM-induced COX2 expression, in HaCaT cells. As illustrated in Fig. 3A–C, PMs exposure resulted in time-dependent increases in Nox activity and ROS generation. These effects were significantly attenuated after pretreatment with AhRI, apocynin (APO; a Nox2 inhibitor), or N-acetylcysteine (NAC; an ROS scavenger). In addition, transfection with siRNAs specific for AhR, Nox2 (gp91^*phox*^) and p47^*phox*^ significantly decreased ROS generation (Fig. 3D). Based on image fluorescence after staining with a mitochondrial membrane potential dye (tetramethylrhodamine methyl ester; TMRM), mitochondrial membrane potential was not changed after 2 h of PM exposure (Fig. 3C,D), suggesting that mitochondria are not be directly involved in PM-associated ROS generation at this time point. In order to examine PM-induced ROS production in HaCaT cells at later time points, the CellRox assay was performed. It can be seen that ROS enhancement occurred at 6, 12 and 24 hours post PM treatment (Supplementary Fig. 1A). We found that long-term ROS production after PM exposure can be decreased by pre-treatment with apocynin, but not by MitoTEMPO (a specific scavenger for mitochondrial superoxide anions) (Supplementary Fig. 1B). On the other hand, we also determined mitochondrial membrane potential using JC-1 dye. As illustrated in Supplementary Fig. 1C, there was a mild loss in mitochondrial membrane potential (loss rate around 10–20%) following PM treatment of HaCaT cells for 6, 12 and 24 hours, but such effects were not attenuated by MitoTEMPO (Supplementary Fig. 1D). Therefore, mitochondrial ROS may not play a major role in mitochondrial membrane potential loss following long-term PM treatment. Moreover, pretreatment with APO or NAC strongly inhibited PM-induced increases in COX2 protein and gene expression, and PGE2 production (Fig. 3E–G). Activation of the Nox2 enzyme requires that the cytosolic component, p47^*phox*^, is phosphorylated and translocated to the plasma membrane with the p67^*phox*^ component, where they assemble into an active enzyme complex^29^. Consistent with this mechanism, we found that PM-induced phosphorylation of p47^*phox*^ occurred in a time-dependent manner (Fig. 3H), with translocation of p47^*phox*^ from the cytosol to the plasma membrane occurring within 15 min (Fig. 3I). In addition, pretreatment with diphenyleneiodonium (DPI), APO or AhRI significantly attenuated PM-induced phosphorylation of p47^*phox*^ located in the plasma membrane (Fig. 3J). These results, obtained using HaCaT cells, suggest that PM-induced up-regulation of COX2 expression is mediated via Nox2/p47^*phox*^-dependent generation of ROS.

### Involvement of ROS-dependent ERK1/2, p38 and JNK1/2 pathways in the induction of COX2 by PM

**Figure 4:**
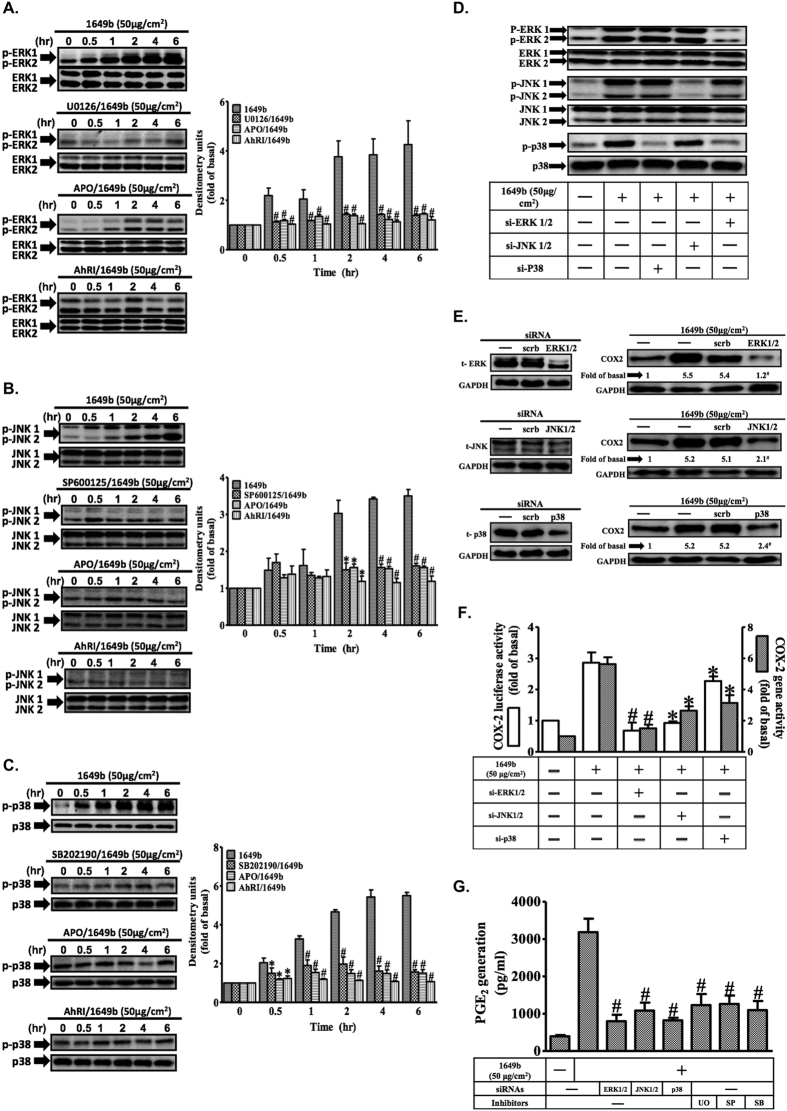
Reactive oxygen species (ROS)-dependent MAPK activation is involved in PMs-induced up-regulation of COX2 expression in HaCaT cells. Cells were pre-incubated with AhRI (10 μM), APO (100 μM), U0126 (10 μM), SB202190 (10 μM), SP60015 (10 μM) or DPI (10 μM) for 1 h and then exposed to PMs (50 μg/cm^2^) for the indicated times (0, 30′, 1 h, 2 h, 4 h and 6 h). (**A–C**) Effects of MAPK inhibitors and antioxidants on phosphorylation of p-ERK1/2 (**A**) p-JNK1/2 (**B**) and p-p38 (**C**). Total levels of each kinase were used as loading controls. Effects of siRNAs or inhibitors of ERK1/2, JNK1/2 and p38 on the phosphorylation of MAPKs (**D**) COX2 protein expression (**E**) COX2 gene expression (**F**) and prostaglandin E2 (PGE2) production (**G**). To assess gene expression (**F**), cells were co-transfected with a COX2 reporter gene and following addition of siRNAs, exposed to PMs for 4 h (for promoter activity evaluations; open bars) or 6 h (for evaluations of mRNA levels; shaded bars). Representative results from three separate experiments are shown. ^*^P < 0.05, ^#^*P* < 0.01, compared with cells exposed to PM alone.

PMs induced phosphorylation of extracellular-signal-regulated kinases 1 and 2 (ERK1/2), p38 and JNK1/2 in a time-dependent manner, and this was significantly attenuated by APO and AhRI (Fig. 4A–C). To further investigate the roles of the three mitogen-activated protein kinase (MAPK) pathways and the interplay between them, siRNAs were used to suppress ERK1/2, p38 and JNK1/2 expression, and the effects on each pathway were evaluated (Fig. 4D). PM-induced phosphorylation of ERK1/2, p38 and JNK1/2 was blocked by transfection with specific siRNAs targeted at each of these signaling components. Inhibition of ERK1/2 signaling with ERK1/2-targeted siRNA caused a significant decrease in p38 phosphorylation, indicating that the ERK1/2 pathway is needed to activate the p38 pathway. In addition, siRNA transfection significantly attenuated PM-induced increases in COX2 protein (Fig. 4E) and gene expression (Fig. 4F), and PGE2 production (Fig. 4G). Dimethyl sulfoxide (DMSO), used as a solvent control, had no significant effect (data not shown). These results, obtained in HaCaT cells, indicate that ROS play a key role in PM-induced ERK1/2, p38 and JNK1/2 phosphorylation that leads to up-regulation of COX2 expression.

### PMs induce COX2 expression via the ROS/ERK/p38 cascade and NF-κB activation

**Figure 5:**
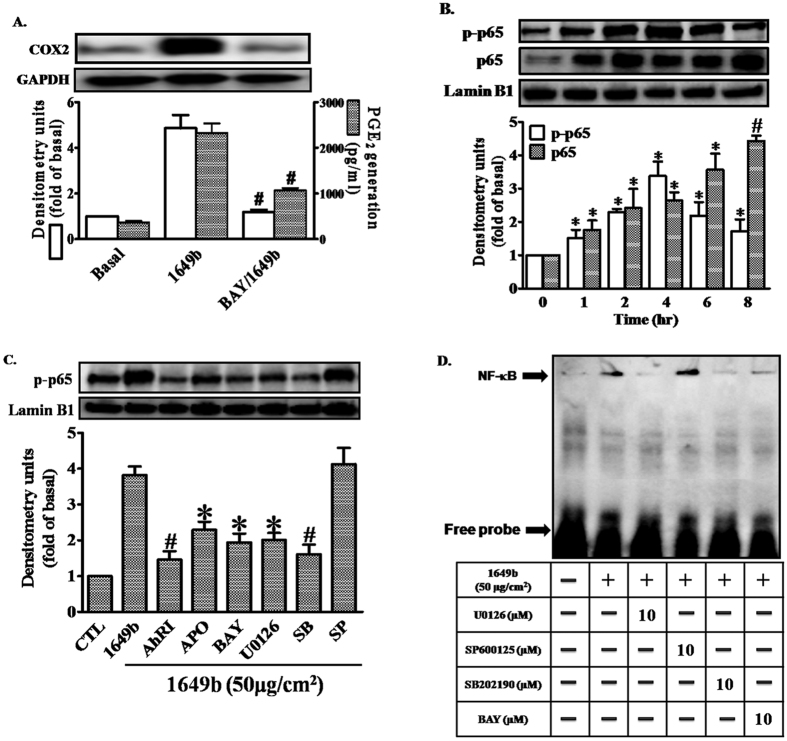
PMs induce COX2 expression via Nox2/ERK/p38/NF-κB signaling. Cells were pre-incubated with NF-κB inhibitor (Bay11-7082; BAY) (10 μM) for 1 h and then exposed to PMs (50 μg/cm^2^) for 24 h. (**A)** COX2 protein expression (open bars) and prostaglandin E2 (PGE2) production (shaded bars) were determined by Western blotting and ELISA as described in Fig. 1. (**B,C**) Cells were pre-incubated with with AhRI (10 μM), APO (100 μM), U0126 (10 μM), SB202190 (10 μM), SP60015 (10 μM) or BAY (10 μM) for 1 h and then exposed to PMs (50 μg/cm^2^) for the 2 h. The levels of p65 in nuclear extract (detected with p65 or phospho-p65 antibodies), determined by Western blotting **(C)**. Lamin-B1 was used as the loading control. (**D)** Cells were pre-incubated with with U0126 (10 μM), SB202190 (10 μM), SP60015 (10 μM) or BAY (10 μM) for 1 h and then exposed to PMs (50 μg/cm^2^) for 2 h. Electrophoretic mobility-shift assay for assessment of NF-κB DNA binding activity, as described in “Materials and methods”. (**A–D**) Data are expressed as mean ± standard error of the mean, based on three independent experiments. ^*^P < 0.05, ^#^*P* < 0.01, compared with basal levels.

Pretreatment of HaCaT cells with an NF-κB inhibitor, Bay11-7082 (BAY), inhibited PM-induced up-regulation of COX2 expression and PGE2 production (Fig. 5A). To determine whether p65 was phosphorylated and translocated to the nucleus in HaCaT cells after exposure to PMs, whole cell lysates and nuclear fractions were prepared. PMs induced rapid phosphorylation and translocation of p65 in HaCaT cells, and this was significantly attenuated after pretreatment with AhRI, APO, BAY, U0126 or SB202190, but not SP600125 (Fig. 5B,C). NF-κB activation by PMs was further confirmed by gel-shift assays; NF-κB binding activity was low in control cells not exposed to PMs, and activity significantly increased after exposure to PMs (Fig. 5D). Pretreatment with BAY, U0126 or SB202190, but not SP600125, blocked the PM-stimulated increase in NF-κB binding activity. These results, obtained in HaCaT cells, demonstrate that NF-κB is involved in the up-regulation of COX2 expression mediated through AhR/ROS generation, which leads to the activation of ERK/p38.

### PMs induce COX2 expression via ROS/JNK signaling and AP-1 activation

**Figure 6:**
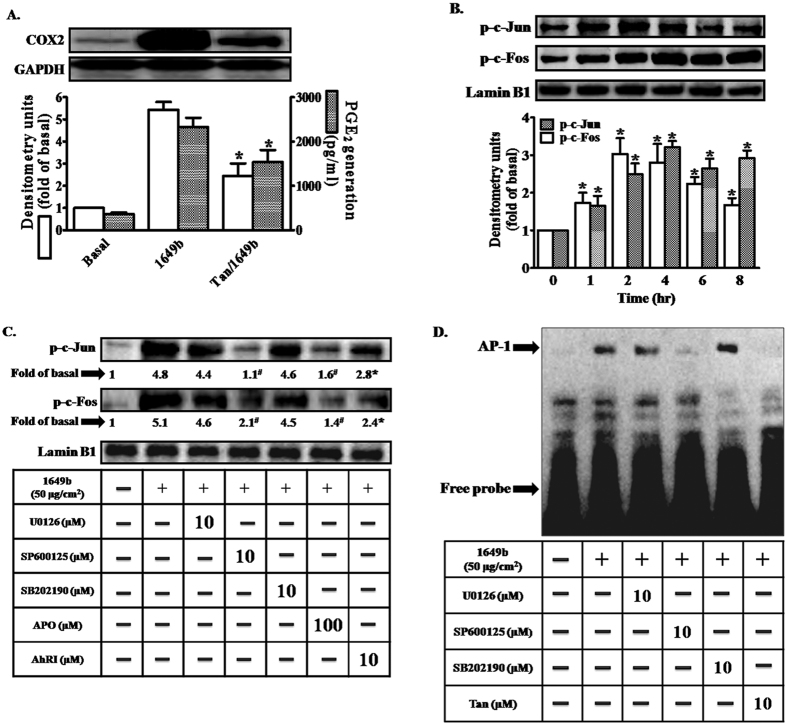
Activation of activator protein 1 (AP-1) is a critical event in PMs-induced up-regulation of COX2 expression. Cells were pre-incubated with AP-1 inhibitor (tanshinone IIA; Tan) (10 μM) for 1 h and then exposed to PMs (50 μg/cm^2^) for 24 h. (**A)** COX2 protein expression (open bars) and prostaglandin E2 (PGE2) production (shaded bars) were determined by Western blotting and ELISA as described in Fig. 1. **(B,C**) Cells were pre-incubated with AhRI (10 μM), APO (100 μM), U0126 (10 μM), SB202190 (10 μM), SP60015 (10 μM) or BAY (10 μM) for 1 h and then exposed to PMs (50 μg/cm^2^) for 2 h. The nuclear extract levels of c-Jun and c-Fos protein were detected using phospho-c-Jun and phospho-c-Fos antibodies, determined by Western blotting (Fig. 6**C**). Lamin-B1 was used as the loading control. (**D)** Cells were pre-incubated with U0126 (10 μM), SB202190 (10 μM), SP60015 (10 μM) or Tan (10 μM) for 1 h and then exposed to PMs (50 μg/cm^2^) for 2 h. Electrophoretic mobility-shift assay for assessment of AP-1 DNA binding activity, as described in “Materials and methods”. Data are expressed as mean ± standard error of the mean, based on three independent experiments. ^*^P < 0.05, ^#^*P* < 0.01, compared with basal levels.

Although ROS and JNK signaling are involved in PM-induced COX2 expression, our results indicate that the activation of NF-κB was not due to inhibition of the JNK pathway. As shown in Fig. 6A, pretreatment of HaCaT cells with tanshinone IIA (an inhibitor of activator protein 1 [AP-1]) attenuated PM-induced increases in COX2 expression and PGE2 production, indicating that AP-1 is required for PM-induced up-regulation of COX2. The AP-1 transcriptional complex comprises at least two major factors: c-Jun and c-Fos. PMs induced phosphorylation of c-Jun and c-Fos in HaCaT cells (Fig. 6B). Pretreatment with the inhibitors AhRI, APO and SP600125 (Fig. 6C), but not U0126 or SB202190, led to significant decreases in PM-induced phosphorylation of both c-Fos and c-Jun. Moreover, increases in AP-1 binding activity following PM exposure (as revealed by gel-shift assays) were significantly attenuated by tanshinone IIA and SP600125, but not U0126 or SB202190 (Fig. 6D). These results suggest that activation of AP-1 signaling, mediated via mechanisms dependent on AhR, ROS and JNK, may be involved in PM-induced up-regulation of COX2 expression in HaCaT cells.

### PMs downregulate filaggrin expression in HaCaT cells by activation of AP-1 and NF-κB pathways

The involvement of the AP-1 and NF-κB pathways in PMs-induced filaggrin downregulation in HaCaT cells was evaluated by Western blotting. As shown in Supplementary Fig. 3, treatment of HaCaT cells with PM (1649b) suppressed filaggrin expression, while pre-treatment with Bay117082 (NF-κB inhibitor) or Tanshinone IIA (AP-1 inhibitor) increased filaggrin expression in PM-stimulated cells. These results indicate that PM may inhibit filaggrin expression by mediating the NF-κB and AP-1 pathways.

### PMs induce IL-6 expression in HaCaT keratinocytes

It has been previously reported that environmental toxic stressors and inflammatory cytokines may play important roles in filaggrin downregulation in keratinocytes^30^,^31^,^32^. We have also performed further flow cytometry and ELISA experiments to determine the protein expression of pro-inflammatory cytokines in HaCaT cells following PMs exposure. As seen in Supplementary Fig. 2, treatment with PM (1649b) induced the expression of IL-6 in HaCaT cells, but had no significant effect on the expression of IL-24, IL-1β or TNF-α. Therefore, our results suggest that PMs exposure may upregulate IL-6 production and subsequently promote inflammatory responses.

### Attenuation of COX-2 expression and filaggrin downregulation in epidermal keratinocytes of PM-exposed mice by blockade of the AhR/NOX pathway

**Figure 7:**
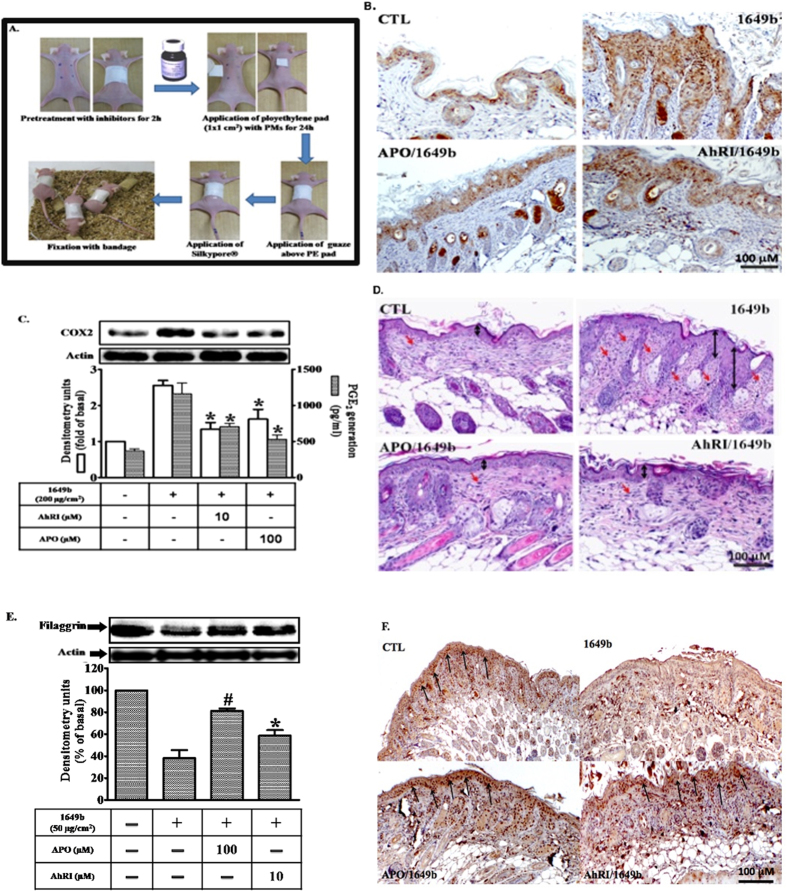
Particulate matters (PMs)-induced cutaneous inflammation was abolished after pre-topical application of an aryl hydrocarbon receptor antagonist (AhRI) and a Nox inhibitor *in vivo*. Mice were anesthetized and received (by pre-topical application) AhRI (10 μM), apocynin (APO; 100 μM) or PGE400 (vehicle-only) for 2 h, after which PMs were topically applied on the dorsal skin (100 μg/cm^2^). After 24 h, mice were sacrificed and skin from the dorsal layer was dissected. (**A**) Procedures for application of PMs to the dorsal skin of mice. (**B,F)** Immunohistochemical staining was performed to assess filaggrin and COX2 expression in the dorsal skin. A brown color indicated filaggrin or COX2-positive cells. Skin sections were observed under a microscope and images were acquired at 400x magnification. (**C,E)** Tissue protein extracts were collected to determine filaggrin or COX2 expression (open bars) by Western blotting and prostaglandin E2 (PGE2) production by ELISA (shaded bars). Representative results from three separate experiments are shown. (**D**) Dorsal skin sections stained with hematoxylin-eosin.

The dorsal skin of mice was treated for 120 min by topical application with vehicle only (PEG400), AhRI (10 μM) or APO (100 μM), 24 h before topical application of PMs. Topical application of PMs is illustrated in Fig. 7A. Immunohistochemical staining was performed to analyze the expression of COX2 and filaggrin in dorsal skin sections of mice. As shown in Fig. 7B,F, expression of COX2 was weak and filaggrin staining was strong (brown color) in the epidermis of the control group. In contrast, COX2 staining was intense in the epidermis of PM-treated mice, but the filaggrin staining was weak. PEG400 had no effect (data not shown). Pre-topical application with AhRI or apocynin attenuated PMs-induced changes in the expression of COX2 and filaggrin in the dorsal skin of mice. Similar effects were observed in protein extracts of mice epidermis (Fig. 7C–E). Histological examination showed PMs-induced thickening of the epidermis, increased hemorrhage in the epidermis, and a greater number of infiltrating inflammatory cells (Fig. 7D). Therefore, PMs down-regulate filaggrin expression in the dorsal skin of mice through an AhR/Nox2/ROS/COX2 mediated mechanism, which may be a central pathway through which PMs participates in skin inflammation and barrier dysfunction.

## Discussion

**Figure 8:**
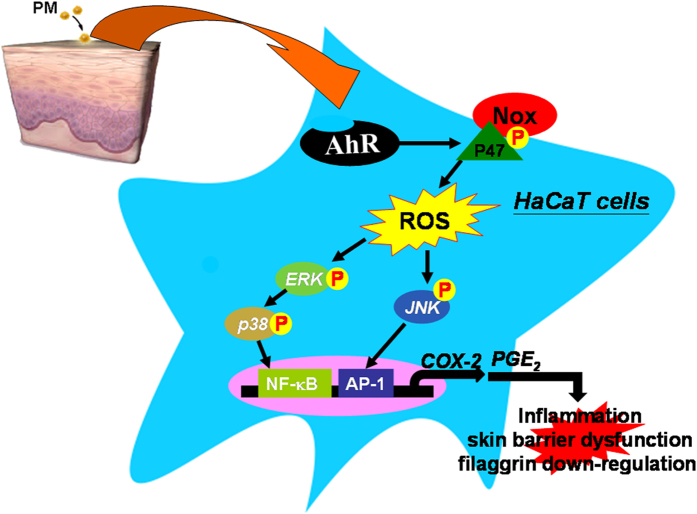
Schematic diagram illustrating the proposed signaling pathway involved in particulate matter (PM)-induced cyclooxygenase 2 (COX-2) expression in HaCaT cells. PMs activate the aryl hydrocarbon receptor (AhR)/Nox2/p47^*phox*^ pathway, which increases generation of reactive oxygen species (ROS), and in turn activates two separate signaling cascades; one involving ERK/p38-dependent activation of NF-κB and another involving JNK-dependent activation of activator protein 1 (AP-1) transcription factors in HaCaT cells. Impairment of skin barrier function by down-regulation of filaggrin via COX2/PGE2 expression in HaCaT cells.

This study demonstrated interactions between COX2/PGE2 and filaggrin in the development of skin barrier dysfunction, using cultures of human keratinocyte cell line. We provide evidence that PMs trigger significant and dose-dependent increases in COX2 protein levels, mRNA expression, promoter activity and PGE2 production, ultimately resulting in filaggrin down-regulation. Our results in human keratinocytes indicate that PM-induced up-regulation of COX2 expression is dependent on activation of the AhR/Nox2/p47^*phox*^ pathway, which is linked to increased ROS generation, and which activates two distinct signaling cascades, ERK/p38/NF-κB and JNK/AP-1 (Fig. 8). Moreover, pre-topical application of AhR antagonists or antioxidants *in vivo* (in mice) protected the skin against PM-induced inflammatory responses by inhibiting COX2 expression, PGE2 production and filaggrin down-regulation.

Using siRNA knock down and AhR antagonists, we showed that AhR activation is required for PM-induced up-regulation of COX2 expression, PGE2 production and filaggrin down-regulation in HaCaT cells. These results are consistent with a recent study, which demonstrated that novel agents that suppress activation of the AhR have protective effects against UV-induced inflammation in human skin^33^. However, there is also accumulating evidence that activation of AhR signaling is beneficial for reducing the severity of inflammatory skin diseases^34^,^35^. Therefore, it is still unclear if AhR inactivation can inhibit inflammation and COX2 expression. The AhR and ROS are implicated in the initiation of various inflammatory responses to PM exposure^36^,^37^. Several reports have shown that HaCaT cells constitutively express Nox1, Nox2, Nox4 and Nox5, as well as the Nox components Rac1, p22^*phox*^, p40^*phox*^, p47^*phox*^ and p67^*phox*^ 38,39,40,41. These Nox isoforms are major contributors to ROS generation in the skin^42^. Here, we show that siRNA-mediated suppression of Nox2 and p47^*phox*^ expression blocked PM-induced up-regulation of COX2 expression and attenuated PGE2 production (Fig. 2B–D). These effects were not observed for siRNAs of Nox1 and Nox4 (data not shown). Our results suggest that PM-induced up-regulation of COX2 expression is mediated through Nox2-dependent ROS generation. We further demonstrated that after PM exposure, both Nox activity and ROS generation were significantly increased, and that these effects were attenuated by pretreatment with AhRI, a Nox2 inhibitor a ROS scavenger. Confocal microscopy also provided strong evidence for accumulation of intracellular ROS after PM exposure. Analysis of image fluorescence indicated significant decreases in ROS generation after transfection with siRNAs for AhR, Nox2 (gp91^*phox*^) and p47^*phox*^.

Using the dye TMRM, we found that an increase in red fluorescence was not changed after 2 h of PM exposure, suggesting that mitochondria are not directly involved in PM-induced ROS generation (Fig. 3C,D). These results are consistent with a previous study that investigated the effect of PMs on human bronchial epithelial cells^43^. In our study, we found that PM treatment promoted ROS generation and subsequently induced mitochondrial membrane potential loss in HaCaT cells at later time points. However, mitochondrial-derived ROS may not play a major role in mitochondrial membrane potential loss as illustrated in Supplementary Fig. 1. Furthermore, we demonstrated that p47^*phox*^ translocates from the cytosol to the plasma membrane within 15 minutes of PM exposure, and that phosphorylation of p47^*phox*^ acts as a signal for Nox2 complex assembly at the membrane. Furthermore, PM-induced phosphorylation of p47^*phox*^ was blocked by pretreatment with AhRI or antioxidants, both within the cell membrane and in the cytosol. In this study, we established for the first time (in HaCaT cells) that Nox2/p47^*phox*^-derived ROS contribute to PM-induced up-regulation of COX2 expression.

We found that PM-stimulated phosphorylation of ERK1/2, JNK1/2 and p38 is due to AhR-mediated induction of Nox2-derived ROS (this was confirmed using the inhibitors apocynin and AhRI). In addition, pretreatment with inhibitors specific for three different MAPK pathways–ERK1/2, JNK1/2 and p38–or transfection with siRNAs specific for components of these pathways–significantly attenuated PM-induced increases in COX2 protein expression (Fig. 4F–H), gene expression (Fig. 4I) and PGE2 production (Fig. 4J). These findings indicate an essential role for these pathways in PM-induced responses. Moreover, we found that inhibition of the ERK1/2 signaling pathway with ERK1/2 siRNA was sufficient to cause a significant decrease in p38 phosphorylation, indicating that the ERK1/2 pathway is necessary to activate the p38 pathway. Distinct groups of MAPKs are activated in an ROS-dependent manner, which is cell type-specific and dependent on external stimuli^44^. This is the first study (performed using HaCaT cells) to describe these kinds of interactive effect between the three MAPK pathways in PM-induced up-regulation of COX2 expression. These results indicate that in HaCaT cells, PMs act via the AhR/Nox2/p47^*phox*^ pathway to increase ROS generation. In turn, this activates two distinct pathways (respectively involving ERK/p38 activation and JNK1/2 phosphorylation), which leads to up-regulation of COX2 expression.

We provide clear evidence that activation of p65 (a subunit of NF-κB) and c-Jun/c-Fos (the dimers of AP-1) are involved in PM-induced up-regulation of COX2 expression (Figs 5B and 6B). Moreover, gel-shift assays and analysis of nuclear fractions directly showed that PMs stimulated binding of NF-κB and AP-1 to the COX2 promoter (Figs 5D and 6D). Suppression of the ERK/p38 and JNK pathways with specific inhibitors significantly attenuated PM-induced promoter-binding of NF-κB and AP-1. Therefore, we conclude that PMs bind to the AhR and activate ERK/p38 and JNK signaling through Nox/ROS-dependent mechanisms. We propose that this induces nuclear translocation and activation of NF-κB and AP-1 phosphorylation.

We demonstrated *in vivo* that the inhibitor AhRI, as well as apocynin (a specific inhibitor of Nox2), suppressed PM-induced inflammatory responses in skin by inhibiting COX-2 expression and PGE2 production. Consistent with these findings, histological examinations showed that PM exposure led to thickening of the epidermis, increased hemorrhage, and higher numbers of infiltrating inflammatory cells; these effects were attenuated after inhibitor treatment (Fig. 7D). PM-induced reductions in filaggrin expression could be reversed by treatment with a selective COX2 inhibitor or a PGE2 receptor antagonist. This suggests that PMs increase PGE2 production by inducing COX2 expression in human keratinocytes, and that up-regulation of PGE2 is associated with PM-induced reductions in filaggrin protein levels.

Recently, Jin *et al*. demonstrate that up-regulation of inflammatory cytokines may play an important role in filaggrin downregulation in keratinocytes^30^. As illustrated in Supplementary Fig. 2, we found that PM (1649b) induced the expression of IL-6 in HaCaT cells, but had no significant effect on the expression of IL-24, IL-1β or TNF-α, which is consistent with previous studies showing an inverse relationship between IL-6 and filaggrin expression^31^,^32^. The environmental toxic stressors used by Jin *et al*.^30^ consisted of chemical irritants (sodium lauryl sulfate) and ultraviolet B. In contrast, the PM (1649b) used in our study was composed of polycyclic aromatic hydrocarbons, polychlorinated biphenyl congeners, chlorinated pesticides, and other atmospheric particulate material. Therefore, the environmental stressors used in our study and the study by Jin *et al*. were very different. This may provide an explanation for the differences in expression profiles of inflammatory cytokines in the two studies, particularly with regards to IL-24 and IL-6. However, the mechanisms need to be further investigated.

Together with several recent studies reporting that increased COX2 expression/PGE2 production can induce skin inflammation^22^,^23^,^45^, our findings further demonstrate the interrelationships between PM exposure, up-regulation of COX2 expression/PGE2 production, down-regulation of filaggrin and skin inflammation. In contrast, adverse effects of the PM standard reference material 1648a, primarily containing heavy metals, were negligible in the stratum corneum and tight junction^27^. This indicates that PM components, and dose, time of analysis and active sides may influence the results.

In conclusion, this is the first study to demonstrate a critical role for AhR/Nox2 signaling in PM-induced up-regulation of COX2 expression and PGE2 production, *in vitro* and *in vivo*. Our results indicate that increased COX2 expression and PGE2 production leads to down-regulation of filaggrin expression in human keratinocytes, which may act as a central pathway through which PMs disrupt skin barrier function. Future development of novel therapies targeted at the AhR will be highly important in strategies to protect against PM-induced skin barrier dysfunction in humans.

## Methods

### Cell cultures and reagents

Human epidermal keratinocytes (HaCaT cells) were generously provided by Professor Feng-Lin Yen (Department of Fragrance and Cosmetic Science, Kaohsiung Medical University, Kaohsiung, Taiwan). Cells were cultured in Dulbecco’s Modified Eagle Medium/Nutrient Mixture F-12 (DMEM/F-12) (GIBCO; Grand Island, NY, USA) supplemented with 10% fetal bovine serum (FBS) (Hazelton Research Products; Denver, PA, USA) and 1% penicillin–streptomycin at 37 °C in 5% CO_2_. When the cultures reached confluence, cells were treated with 0.05% (w/v) trypsin/0.53 mM EDTA for 5 min at 37 °C. The cell suspension was then diluted with DMEM/F-12 containing 10% FBS (Invitrogen; Carlsbad, CA, USA) to a concentration of 2 × 10^5^ cells/ml, and plated onto 12-well culture plates (1 ml/well) and 10-cm culture dishes (10 ml/dish) for measurement of protein expression and mRNA accumulation, respectively. Experiments were performed using cells between passages 5–12.

The characteristics of the PMs (SRM 1649b, obtained from NIST; Gaithersburg, MD, USA) have been described previously^27^. Monoclonal antibodies against COX2, filaggrin, p47^*phox*^, gp91^*phox*^, β-actin, anti-phospho c-Fos and anti- phospho c-Jun were obtained from Santa Cruz Biotechnology (Santa Cruz, CA, USA). PhosphoPlus p42/p44 MAPK, p38MAPK and SAPK/JNK antibodies were obtained from Cell Signaling (Danvers, MA, USA). PhosphoPlus p47^*phox*^ antibody was obtained from Assay Biotechnology (Sunnyvale, CA, USA). Anti-glyceraldehyde 3-phosphate dehydrogenase (GAPDH) antibody was obtained from Biogenesis (Bournemouth, UK). The COX2 inhibitor, NS398, and the EP1/EP2 receptor antagonist, AH-6809, and MitoTEMPO (a specific scavenger for mitochondrial superoxide anions) were obtained from Cayman (Ann Arbor, MI, USA). N-acetylcysteine (NAC), apocynin (APO), Bay11-7082 (BAY), tanshinone IIA, U0126, SB202190 and SP600125 were obtained from Biomol (Plymouth Meeting, PA, USA). The AhR antagonist was obtained from Calbiochem (San Diego, CA, USA). The chloromethyl derivative of H_2_DCFDA (CM-H_2_DCFDA) was obtained from Molecular Probes (Eugene, OR, USA). The bicinchoninic acid (BCA) protein assay kit was obtained from Pierce (Rockford, IL, USA). JC-1 was purchased from Sigma-Aldrich (St. Louis, MO, USA). CellROX™ green reagent (C10444) was purchased from Life Technologies. Enzymes and other chemicals were obtained from Sigma (St. Louis, MO, USA).

### Preparation of cell extracts and Western blot analysis

HaCaT cells were plated onto 12-well culture plates and made quiescent at confluence by incubation in serum-free DMEM/F-12 for 24 h. Growth-arrested cells were incubated with or without PMs (at various concentrations and durations) at 37 °C. For inhibition experiments, inhibitors were added 1 h prior to the application of PMs. After incubation, the cells were rapidly washed in ice-cold phosphate-buffered saline (PBS), scraped and then collected by centrifugation at 1000 g for 10 min. The collected cells were lysed with ice-cold lysis buffer containing 25 mM Tris-HCl (pH 7.4), 25 mM NaCl, 25 mM NaF, 25 mM sodium pyrophosphate, 1 mM sodium vanadate, 2.5 mM EDTA, 0.05% (w/v) Triton X-100, 0.5% (w/v) sodium dodecyl sulfate (SDS), 0.5% (w/v) deoxycholate, 0.5% (w/v) NP-40, 5μg/ml leupeptin, 5μg/ml aprotinin, and 1 phenylmethylsulfonyl fluoride (PMF). Lysates were centrifuged at 45,000×g for 1 h at 4 °C to yield the whole cell extract. Protein concentration was determined using BCA reagents, according to the manufacturer’s instructions. Samples from the supernatant fractions (30 μg protein) were denatured and subjected to SDS-PAGE using a 10% running gel. Proteins were then transferred to nitrocellulose membranes, which were incubated successively at room temperature with 5% (w/v) bovine serum albumin (BSA) in Tris-buffered saline with Tween-20 (TTBS) (50 mM Tris-HCl, 150 mM NaCl, 0.05% (w/v) Tween 20, pH 7.4) for 1 h. Membranes were incubated overnight at 4 °C with various antibodies, using dilutions of 1:1000 in TTBS. After washing in TTBS four times (5 min each), membranes were incubated with anti-rabbit or anti-mouse horseradish peroxidase antibodies (1:2000 dilutions) for 1 h. After antibody incubations, membranes were washed thoroughly in TTBS. Immunoreactive bands detected by ECL reagents were developed using Hyperfilm-ECL. DMEM/F-12 was purchased from Invitrogen (Carlsbad, CA, USA). Hybond C membranes, the enhanced chemiluminescence (ECL) Western blotting detection system and Hyperfilm were obtained from GE Healthcare Biosciences (Buckinghamshire, UK).

### Preparation of particle matter samples

SRM 1649b suspensions were prepared in cell culture PBS at a particle matter (PM) concentration of 1000 μg/ml (mass/volume).The suspended particles were then sonicated for 30 min to avoid agglomeration. Experiments were performed within 1 h of PM preparation to avoid variability in PM components in different fractions between replicates. Cells were exposed to the total PM suspensions at concentrations of 10 to 1000 μg/ml for 24 h. PM suspensions were prepared in PBS for bioactivity assays.

### Quantitative real-time RT-PCR

Total RNA was isolated with TRIzol (Invitrogen; Carlsbad, CA, USA), according to the manufacturer’s protocol. cDNA obtained from 0.5 μg total RNA was used as a template for PCR amplification, as previously described^22^. The reverse transcriptase reaction was carried out using Moloney murine leukemia virus reverse transcriptase (Invitrogen). Total RNA was added to a reaction mixture containing oligo-deoxythymidine (0.5 mg/ml), deoxynucleotide (dNTP) (20 mM), dithiothreitol (0.1 M), Tris-HCl (250 mM, pH 8.3), KCl (375 mM), and MgCl2 (15 mM). The reaction was carried out at 37 °C for 90 min. Quantitative real-time PCR was performed in a reaction mixture containing 1X Smart Quant Green Mix, cDNA (50 ng), and the following primers using MX3000P (Stratagene): 5′-GTAACCCGTTGAACCCCATT-3′ (forward) and 5′-CCATCCAATCGGTAGTAGCG-3′ (reverse) for 18 s ; 5′-TGCATTCTTTGCCCAGCACT-3′ (forward) and 5′-AAAGGCGCAGTTTACGCTGT-3′ (reverse) for COX2;

### RT-PCR analysis

Total RNA was isolated with Trizol according to the protocol of the manufacturer. The cDNA obtained from 0.5 μg total RNA was used as a template for PCR amplification as previously described^46^. The primers used were as follows: 5′-TGACGGGGTCACCCACACTGTGCCCATCTA-3′ (sense) and 5′-CTAGAAGCATTTGCGGTGGACGATG-3′ (anti-sense) for β-actin; 5′-TGATGCAGTCTCCCTCTGTG-3′ (sense) and 5′-TGTTTCTCTTGGGCTCTTGG-3′ (anti-sense) for filaggrin.

### Measurement of PGE2 production

HaCaT cells were cultured in 12-well culture plates. After reaching confluence, cells were treated with PMs for different time durations (as indicated). After treatment, medium was collected and stored at −80 °C until assays were performed. PGE2 production was assayed using the PGE2 enzyme immunoassay kit (Cayman), according to the manufacturer’s instructions.

### Measurement of COX2 luciferase activity

The COX2 luciferase reporter gene was generously provided by Professor Lee-Wen Chen (Department of Respiratory Care, Chang Gung Institute of Technology, Chia-Yi, Taiwan). For construction of the COX2-luc plasmid, the human COX2 promoter (spanning the −459 to +9 bp region) was cloned into the pGL3-basic vector (Promega, Madison, WI, USA). COX2-luc activity was determined, as previously described, using a luciferase assay system (Promega; Madison, WI, USA). Approximately 5 μl of the resulting supernatant was mixed with 50 μl luciferase assay solution, and luminescence was determined using a Fluoroskan Ascent FL (Thermo Scientific^®^) luminometer. All experiments were performed in triplicate. Renilla luciferase activities were standardized to Renilla activity.

### Transient transfection with siRNAs

SMARTpool RNA duplexes corresponding to human p47^*phox*^(SC-29422), Nox2 (gp91^*phox*^, SC35503) and scrambled siRNA (SC-37007) were obtained from Santa Cruz Biotechnology (Santa Cruz, CA, USA). JNK1/2 siRNA (SASI_Hs01_00010440), ERK1/2 siRNA (SASI_Hs01_00190617), p38 siRNA (SASI_Hs01_00018464) and AhR siRNA (SASI_Hs01_00093959) were obtained from Sigma (St. Louis, MO, USA). Transient transfection of siRNAs was performed using transfection reagent. siRNAs (100 nM) were formulated with transfection reagent according to the manufacturer’s instructions. The transfection efficiency (approximately 60%) was determined by transfection with enhanced green fluorescent protein (EGFP).

### Isolation of the cell fraction

The cytosolic and plasma membrane fractions were prepared by a modification of the method described previously. Briefly, HaCaT cells were lysed in lysis buffer A (20 mM Tris–HCl, 10 mM EGTA, 2 mM EDTA, 2 mM dithiothreitol, 1 mM PMF, 25 μg/ml aprotinin, and 10 μg/ml leupeptin). Cell lysates were centrifuged at 16,000 g for 20 min at 4 °C. Supernatant was collected and designated the cytosolic fraction. Pellets were re-suspended in lysis buffer B (0.5% sodium dodecyl sulfate, 1% NP-40, 1 mM Na_3_VO_4_, 1 mM NaF, 1 mM PMF, 25 μg/ml aprotinin, and 10 μg/ml leupeptin). Western blot analysis for p47^*phox*^ was performed on the plasma membrane fraction as described above, using a monoclonal mouse antibody against p47^*phox*^.

### Determination of NADPH oxidase activity by chemiluminescence assay

Nox activity in intact cells was assayed by lucigenin chemiluminescence^47^. After incubation, cells were gently scraped and centrifuged at 400×g for 10 min at 4 °C. The cell pellet was re-suspended in ice-cold RPMI 1640 (35 μl/well), and the cell suspension was kept on ice. Five microliters of cell suspension (2 × 10^4^ cells) were added to a final 200-μl volume of pre-warmed RPMI 1640 medium (37 °C) containing either 1 μM NADPH (Sigma) or 20 μM lucigenin (Sigma), in order to initiate the chemiluminescent reaction. Chemiluminescence was measured using a Fluoroskan Ascent FL fluorometer (Thermo Scientific^®^) in the out-of-coincidence mode (appropriate blanks and controls were established). Neither NADPH nor nicotinamide adenine dinucleotide (NADH) enhanced the background chemiluminescence of lucigenin alone (30–40 counts/min). Chemiluminescence was continuously measured for 15 min, and the activity of Nox was expressed as counts per million cells.

### Measurement of intracellular ROS generation

Intracellular H_2_O_2_ levels were determined by measuring fluorescence of DCFH-DA. For these experiments, cells were washed in warm Hank’s balanced salt solution (HBSS) and then incubated in HBSS or cell medium containing 10 μM DCFH-DA at 37 °C for 45 min. Subsequently, HBSS or medium containing DCFH-DA was removed and replaced with fresh medium. Cells were then incubated with various PM concentrations, washed twice with PBS, and detached with trypsin/EDTA. Relative fluorescence intensity was recorded over time (3–120 min) using the Fluoroskan Ascent FL fluorescent plate reader at an excitation wavelength of 485 nm, and emissions were measured at a wavelength of 530 nm.

### Chemicals and loading of fluorescent dyes for measurement of ROS generation

All chemicals were obtained from Sigma (St. Louis, Mo., USA), and fluorescent dyes were purchased from Molecular Probes Inc. (Eugene, Oregon., USA). TMRM was used to measure mitochondrial membrane potential (100 nM) and 6-carboxy-2′,7′-dichlorodihydrofluorescein diacetate (DCF) was used as a ROS probe (500 nM). Fluorescent probes were loaded at room temperature for 20–30 min. After loading, cells were rinsed three times in PBS. Cells loaded with dyes in the ester form (i.e. DCF) required an additional 30 min before use. Dye-loaded cells were mounted in a cell chamber for microscopic observation. Confocal fluorescence images were obtained using a Leica confocal microscope (model TCSNT) equipped with an Ar/Kr laser and filters specific for DCF (excitation wavelength: 450–490 nm; emission wavelength: 515–565 nm) and TMRM (excitation wavelength: 540–550 nm; emission wavelength: LP 590–600 nm). All experiments were performed at room temperature.

### Electrophoretic mobility-shift assay

The preparation of nuclear protein extracts and electrophoretic mobility-shift assay (EMSA) conditions have been described previously^22^,^48^. Nuclear proteins were extracted using NE-PER reagent (Pierce, Rockford, IL, USA), according to the manufacturer’s protocol. AP-1 activity and NF-κB binding activity were analyzed using equal amounts of nuclear protein (10 μg) with a LightShift Chemiluminescent EMSA kit (Pierce). The synthetic double-stranded oligonucleotides used as probes in the gel-shift assay were 5′-AGTTGAGGGGACTTTCCCAGGC-3′ and 3′-TCAACTCCCCTGAAAGGGT CCG-5′ for NF-κB, and 5′-CGCTTGATGAGT-CAGCCGGAA-3′ and 3′-GCGAACTACTC AGTC GGCCTT-5′ for AP-1.

### Mouse model and immunohistochemical staining

Male 8-week-old BALB/c nude mice (n = 24) were purchased from the National Laboratory Animal Center, Taiwan. Animals were cared for in accordance with institutional and international standards (Guide for the Care and Use of Laboratory Animals, NIH), and the protocol for animal procedures was approved by the Institutional Animal Care and Use Committee (IACUC) of the Kaohsiung Medical University (IACUC-102178). Upon arrival, animals were acclimated in a room at controlled temperature (25 °C), humidity (50 ± 10%) and a 12-h day-night cycle (lights on between 07:00–19:00 h) for at least 7 days before experiments started. Animals were housed in groups of two to five per cage and provided with food (Western Lab 7001; Orange, CA, USA) and water *ad libitum*. Mice weighing between 25–30 g were used in experiments, and handled for 3 to 4 days before experiments were started. Mice received vehicle (PEG400), AhRI (10 μM) or apocynin (100 μM), administered topically, for 120 min before PM application. PMs (SRM 1649b) were dispersed in PBS at a concentration of 0.1 mg/ml, spread on a nonwoven polyethylene pad (3 M; Taipei, Taiwan) with an area of 1 cm × 1 cm, and then applied to the dorsal region of mice. The dose of PMs on the skin was 100 μg/m^2^. The pad was fixed using Tegaderm^®^ adhesive dressing (3 M; St. Paul, MN, USA) and gauze, and a Silkypore^®^ elastic adhesive gauze (Alcare; Tokyo, Japan) was used to cover the pad. The cloth was replaced every 24 h. After 5 days of consecutive application, the cloth was removed, and the treated area was cleaned before examination (the procedures for PM application are illustrated in Fig. 7**A**). The skin of the dorsal region was immediately dissected into two parts; one of these parts was post-fixed by 4% paraformaldehyde for immunohistochemistry, and the other was frozen in liquid nitrogen and then stored at −80 °C for subsequent immunoblotting and PGE2 assay procedures. For total protein isolation, skin tissue was homogenized in cold 1× RIPA buffer (FIVEphoton Biochemicals; San Diego, CA, USA) with protease inhibitor cocktail (PI-1; FIVEphoton Biochemicals) to obtain rough tissue lysate. Tissue lysate samples were then sonicated for 3 min (with a 20 s running-10 s resting cycle) on ice to obtain the tissue homogenate. Small aliquots of the extract were retained for protein determination by the Coomassie brilliant blue assay, using BSA as the standard.

### Histological analysis

The dorsal skin was excised from the mice after sacrifice by isoflurane. The skin species were immersed in a 10% buffered formaldehyde using ethanol, embedded in paraffin wax, and sliced at a thickness of 3 mm. The samples were stained by hemoxylin and eosin (H&E) and imaged under light microscopy (IX81, Olympus, Tokyo, Japan). Immunohistochemistry was performed using the Polink-2 HRP Plus Rabbit DAB Detection System (Golden Bridge International, Inc.) according to manufacturer’s recommendations, the primary anti-COX2 (1:500, ab15191, abcam) and anti-filaggrin (1:500, ab24584, abcam) antibodies were incubated with the skin specimens at room temperature for 1 h. The skin sections stained with COX2 and filaggrin were visualized by light microscopy.

### Analysis of data

All data were analyzed using the GraphPad Prism Program (GraphPad, San Diego, CA, USA). Data are expressed as mean ± standard error of the mean (SEM). And analyzed by one-way ANOVA followed by Tukey’s post-hoc test. *P* < 0.05 was considered statistically significant.

## Additional Information

**How to cite this article**: Lee, C.-W. *et al*. Urban particulate matter down-regulates filaggrin via COX2 expression /PGE2 production leading to skin barrier dysfunction. *Sci. Rep.* **6**, 27995; doi: 10.1038/srep27995 (2016).

## Supplementary Material

Supplementary Information
