# Supplementary material for: Urban particulate matter down-regulates filaggrin via COX2 expression/PGE2 production leading to skin barrier dysfunction
Source: Sci Rep. 2016 Jun 17;6:27995. doi: 10.1038/srep27995 (PMC4911555; doi:10.1038/srep27995)
Supplement: Supplementary Information [file srep27995-s1.pdf]

Urban particulate matter down-regulates filaggrin via COX2 expression /PGE2 production leading to skin barrier dysfunction

Chiang-Wen Lee[1,2], Zih-Chan Lin[2,3,4], Stephen Chu-Sung Hu[5,6], Yao-Chang Chiang[7,8], Lee-Fen Hsu[9], Yu-Ching Lin[9,10,11], I-Ta Lee[8], Ming-Horng Tsai[12*], Jia-You Fang[2,3,13*]

[1]*Department of Nursing, Division of Basic Medical Sciences, Chang Gung University of Science and Technology, Chia-Yi, Taiwan.*
[2]*Research Center for Industry of Human Ecology, Chang Gung University of Science and Technology, Kweishan, Taoyuan, Taiwan.*
[3]*Pharmaceutics Laboratory, Graduate Institute of Natural Products, Chang Gung University, Kweishan, Taoyuan, Taiwan.*
[4]*School of Traditional Chinese Medicine, Chang Gung University, Kweishan, Taoyuan, Taiwan.*
[5]*Department of Dermatology, College of Medicine, Kaohsiung Medical University, Kaohsiung, Taiwan.*
[6]*Department of Dermatology, Kaohsiung Medical University Hospital, Kaohsiung, Taiwan.*
[7]*Center for Drug Abuse and Addiction, China Medical University Hospital, Taichung, Taiwan,*
[8]*School of Medicine, College of Medicine, China Medical University, Taichung, Taiwan*
[9]*Department of Respiratory Care, Chang Gung University of Science and Technology, Chiayi Campus, Chiayi, Taiwan.*
[10]*Department of Respiratory Care, Chang Gung University, Taoyuan, Taiwan.*
[11]*Division of Pulmonary and Critical Care Medicine, Chang Gung Memorial Hospital, Chiayi, Taiwan.*
[12]*Department of Pediatrics, Division of Neonatology and Pediatric Hematology/Oncology, Chang Gung Memorial Hospital, Yunlin, Taiwan.*
[13]*Chinese Herbal Medicine Research Team, Healthy Aging Research Center, Chang Gung University, Kweishan, Taoyuan, Taiwan.*

***Correspondence:**
Ming-Horng Tsai, MD, Division of Neonatology and Pediatric Hematology/Oncology, Department of Pediatrics, Chang Gung Memorial Hospital, No. 707 Gongye Road, Sansheng, Mailiao Township, Yunlin, Taiwan, R.O.C. Tel: +886-5-6915151-2893, Fax: +886-5-6913222; E-mail address: mingmin.tw@yahoo.com.tw; Prof. Jia-You Fang, Pharmaceutics Laboratory, Graduate Institute of Natural Products, Chang Gung University, 259 Wen-Hwa 1st Road, Kweishan, Taoyuan 333, Taiwan, Tel: +886-3-2118800, Fax: +886-3-2118236; E-mail: fajy@mail.cgu.edu.tw.

Supplementary Materials & Methods

Reagents

MitoTEMPO (a specific scavenger for mitochondrial superoxide anions) was obtained from Cayman (Ann Arbor, MI, USA). JC-1 was purchased from Sigma-Aldrich (St. Louis, MO, USA). CellROX™ green reagent (C10444) was purchased from Life Technologies. Cytokines assay kit: IL-24 (Assay Biotech #OK-0244); IL-1β (Biolegend # 740119); TNF-α (Biolegend # 740122); IL-6 (Biolegend # 740124); Buffer Set (Biolegend # 740075); Human Inflammation Panel Detection Antibodies (Biolegend # 740132); Human Inflammation Panel Standard (Biolegend # 740400). Bay11-7082 (BAY) and tanshinone IIA were obtained from Biomol (Plymouth Meeting, PA, USA).

Measurement of ROS content by CellRox assay

HaCaT cells were cultured in 12-well plates, and treated with PM for various time intervals. Following treatment, CellROX™ green reagent (C10444) (Life Technologies) was added to HaCaT cells with a final concentration of 5 nM. Subsequently, cells were washed in PBS and harvested, and the fluorescence intensity of the cells was analyzed using a FACScan flow cytometer (BD Biosciences, San Jose, CA, USA) at 495-nm excitation and 529-nm emission for CellROX®.

Determination of mitochondrial membrane potential

HaCaT cells were seeded in 12-well plates at 37˚C and 5% CO_2, and treated with PM for various time intervals. Following treatment, cells were incubated with 10 μg/ml JC-1 (5,5',6,6'-tetrachloro-1,1',3,3'-tetraethylbenzimidazolyl-carbocyanine iodide) at 37˚C for 30 minutes. Subsequently, cell lysates were analyzed using a FACScan flow cytometer (BD Biosciences, San Jose, CA, USA) (excitation wavelength 490 nm, emission wavelength 590 nm for red fluorescence and 540 nm for green fluorescence).

Measurement of inflammatory cytokines expression

Human IL-24 production was determined by ELISA kit in culture supernatants according to the manufacturer's protocol (OK-0244, Assay Biotech), by measuring light absorbance at 450 nm using an ELISA reader. The levels of other cytokines (IL-1β, IL-6 and TNF-α) were determined by **LEGENDplex™** (Biolegend) and analyzed using a FACScan flow cytometer (BD Biosciences, San Jose, CA, USA).

Supplementary Figures

Supplementary Fig. S1

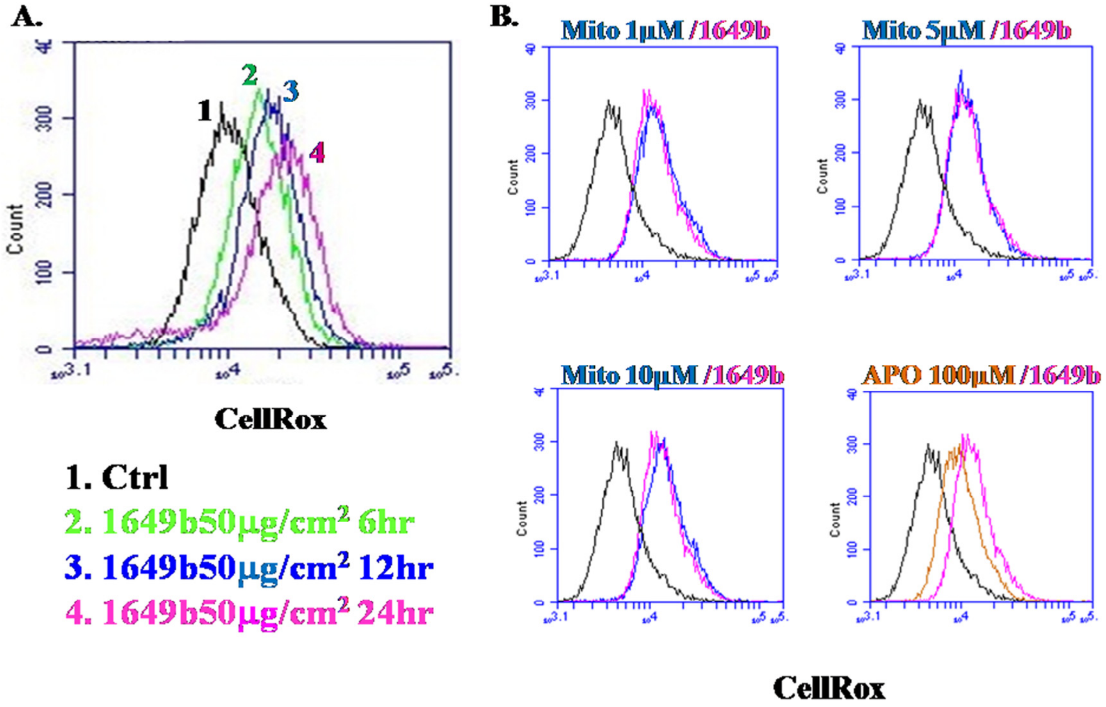

Supplementary Fig. S1

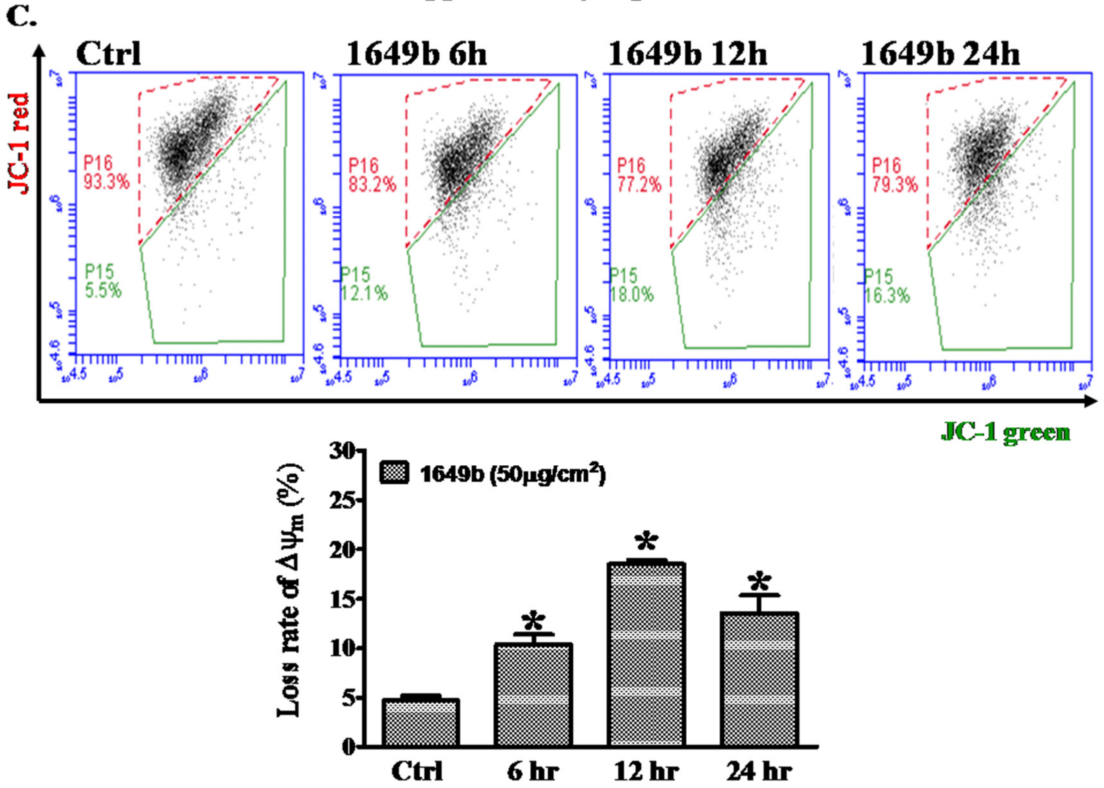

Supplementary Fig. S1

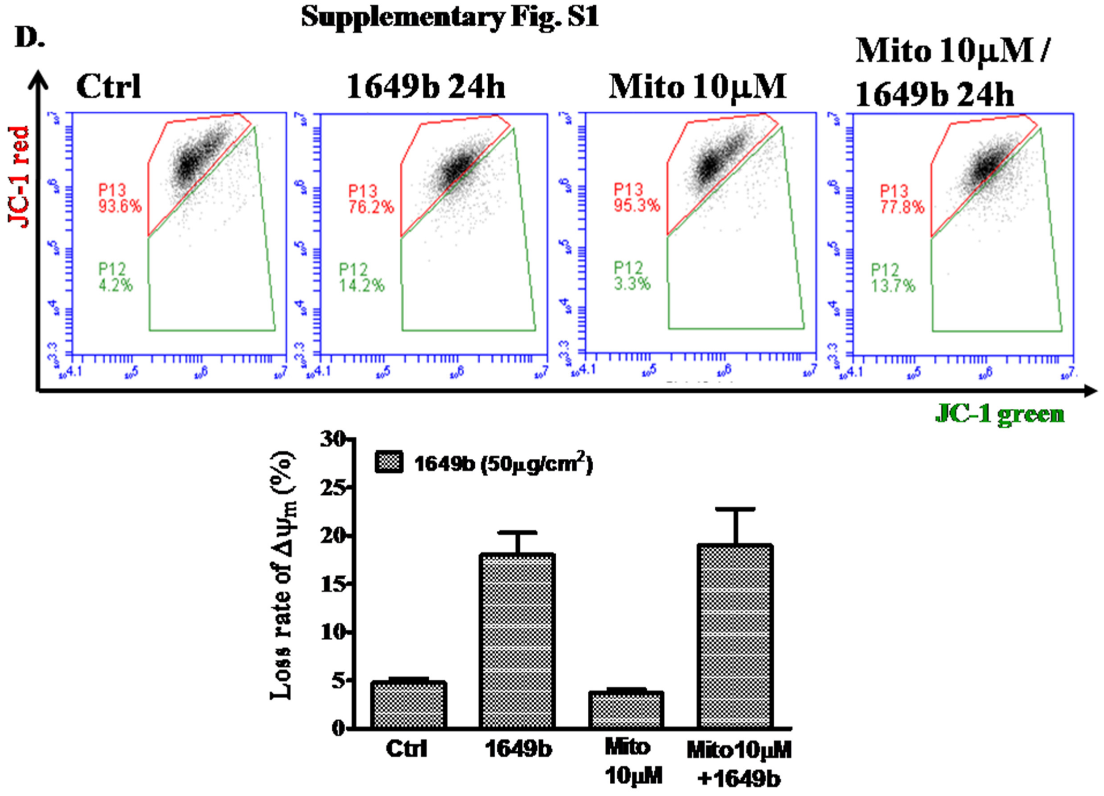

Supplementary Fig. S1. Effect of MitoTEMPO on 1649b particulate matter (PM)-induced long-term production of ROS in HaCaT keratinocytes. (A) HaCaT cells were labeled with CellRox (5 nM) and then treated with 1649b (50 μg/cm^2) for the indicated times (6h, 12h and 24h). **(B)** Confluent cells were labeled with CellRox (5 nM) and then pre-incubated with or without MitoTEMPO (1 μM, 5 μM, 10 μM) and apocynin (100 μM). After incubation for 1h, cells were stimulated with 1649b (50 μg/cm^2) for 24h. The ROS production was measured by flow cytometry. **(C,D)** HaCaT cells were labeled with JC-1 (10 μg/ml) and then pre-treated with or without mitoTEMPO (10 μM). After incubation for 1h, cells were stimulated with 1649b (50 μg/cm^2) for the indicated times (6h, 12h and 24h). The $\triangle\psi_m$ was measured using JC-1 fluorescence dye by flow cytometry. Results are representative of three independent experiments.

Supplementary Fig. S2

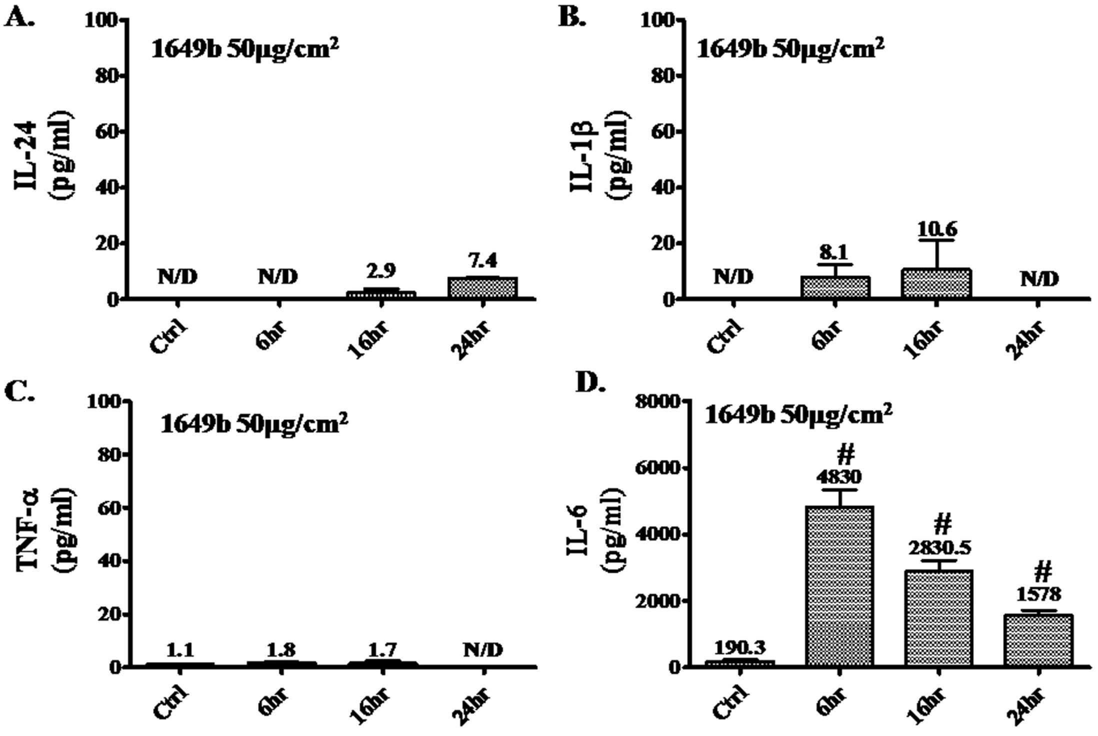

Supplementary Fig. S2. Effect of PM (1649b) stimulation on production of various cytokines in HaCaT keratinocytes. Cells were stimulated with 1649b (50 μg/cm^2) for the indicated times (6h, 16h and 24h), and then the cell culture supernatants were harvested to determine **(A)** IL-24 concentrations by ELISA, **(B-D)** TNF-α, IL-1β and IL-6 concentrations by a flow cytometer. Values represent the mean concentration ± SD of cytokines (n= 3). $^{\#}P < 0.05$.

Supplementary Fig. S3

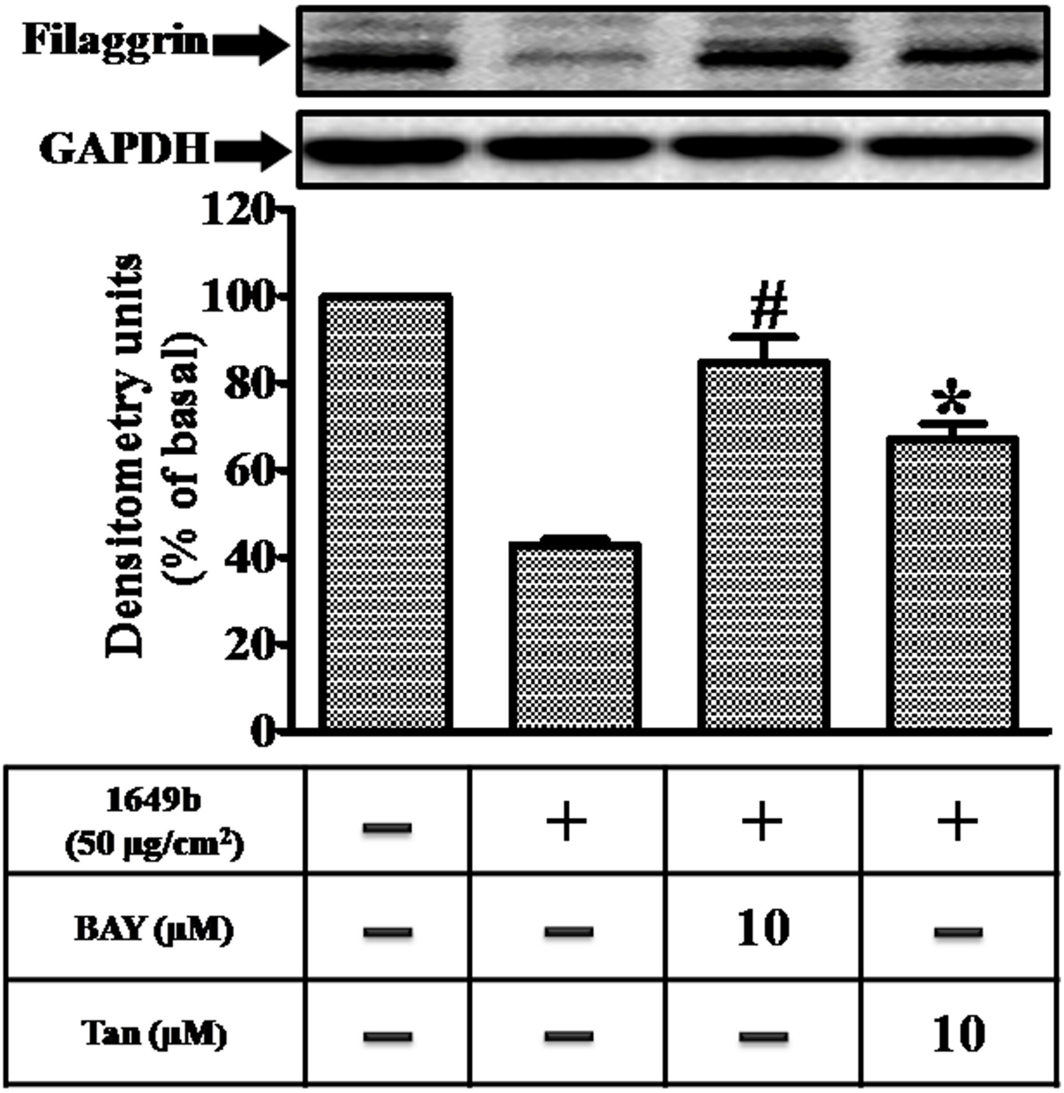

Supplementary Fig. S3. Effects of NF-κB inhibitor (Bay117082, 10 μM) and AP-1 inhibitor (Tanshinone IIA, 10 μM) on PM-induced filaggrin downregulation in HaCaT cells, determined by Western blotting. Data expressed as mean ± standard error of the mean, based on three independent experiments. #*P*<0.05 and **P*<0.01 compared with PM (1649b) treatment alone.
